# The effect of phytoglobin overexpression on the plant proteome during nonhost response of barley (*Hordeum vulgare*) to wheat powdery mildew (*Blumeria graminis* f. sp. *tritici*)

**DOI:** 10.1038/s41598-020-65907-z

**Published:** 2020-06-08

**Authors:** O. A. Andrzejczak, C. K. Sørensen, W.-Q. Wang, S. Kovalchuk, C. E. Hagensen, O. N. Jensen, M. Carciofi, M. S. Hovmøller, A. Rogowska-Wrzesinska, I. M. Møller, K. H. Hebelstrup

**Affiliations:** 1 Department of Agroecology, Aarhus University, Forsøgsvej 1, DK, 4200 Slagelse, Denmark; 2 Department of Biochemistry & Molecular Biology and VILLUM Center for Bioanalytical Sciences, University of Southern Denmark, Campusvej 55, DK, 5230 Odense M, Denmark; 3 Department of Molecular Biology and Genetics, Aarhus University, Forsøgsvej 1, DK, 4200 Slagelse, Denmark; 4 Present Address: Key Laboratory of Plant Molecular Physiology, Institute of Botany, the Chinese Academy of Sciences, 100093 Beijing, China; 5 Present Address: Laboratory of Bioinformatic methods for Combinatorial Chemistry and Biology, Shemyakin-Ovchinnikov Institute of Bioorganic Chemistry, RAS Moscow, Russia

**Keywords:** Proteomic analysis, Biotic, Molecular engineering in plants, Cell wall

## Abstract

Nonhost resistance, a resistance of plant species against all nonadapted pathogens, is considered the most durable and efficient immune system in plants. To increase our understanding of the response of barley plants to infection by powdery mildew, *Blumeria graminis* f. sp. *tritici*, we used quantitative proteomic analysis (LC-MS/MS). We compared the response of two genotypes of barley cultivar Golden Promise, wild type (WT) and plants with overexpression of phytoglobin (previously hemoglobin) class 1 (HO), which has previously been shown to significantly weaken nonhost resistance. A total of 8804 proteins were identified and quantified, out of which the abundance of 1044 proteins changed significantly in at least one of the four comparisons (‘i’ stands for ‘inoculated’)- HO/WT and HOi/WTi (giving genotype differences), and WTi/WT and HOi/HO (giving treatment differences). Among these differentially abundant proteins (DAP) were proteins related to structural organization, disease/defense, metabolism, transporters, signal transduction and protein synthesis. We demonstrate that quantitative changes in the proteome can explain physiological changes observed during the infection process such as progression of the mildew infection in HO plants that was correlated with changes in proteins taking part in papillae formation and preinvasion resistance. Overexpression of phytoglobins led to modification in signal transduction prominently by dramatically reducing the number of kinases induced, but also in the turnover of other signaling molecules such as phytohormones, polyamines and Ca^2+^. Thus, quantitative proteomics broaden our understanding of the role NO and phytoglobins play in barley during nonhost resistance against powdery mildew.

## Introduction

Fungal pathogens are one of the main biotic stress factors affecting the growth and development of plants. Fungi are heterotrophs and use the resources of their host, which can lead to significant reductions in the yield and quality of cultivated plants. With the increasing human population and limited food resources, it is becoming increasingly important to understand the mechanisms of plant resistance to pathogens to be able to breed resistant varieties whether by conventional means or by GMO technologies^1^. Plants are characterized by having a general resistance to most of the pathogens found in nature. This so-called nonhost resistance type of immunity is considered the most innate and durable immune system that efficiently detects potential pathogens and initiates a resistance response^2,3^. Nonhost resistance means that a specific pathogen species is not able to grow on a specific plant species. This is due to the physical (surface structures like the cuticle) and chemical (diverse array of secondary metabolites) defense barriers and a system for detection of so‐called pathogen‐associated molecular patterns (PAMPs) produced by the pathogen^4^. Plants have surface-localized receptors called pattern-recognition receptors (PRR) that can perceive PAMPs or damaged-self signals released during alteration of host cell integrity. These signals induce a resistance response referred to as PAMP-triggered immunity (PTI). The pathogen has to suppress PTI in order to establish a compatible interaction and successfully colonize the plant. To do that, pathogens secrete a number of effector proteins. In turn, plants have evolved a designated effector-triggered immunity (ETI), where intracellular immune receptors, which often belong to the nucleotide-binding domain and leucine-rich repeat-containing (NLR) family, are able to recognize these effectors^5^. Susceptibility (compatibility) results when the individual plant lacks the genes coding for the effector-matching ETI receptors. The mildew pathogen *Blumeria graminis* is among the fungi causing the most severe losses in cereals. During its evolution *B. graminis* has split into different formae speciales (ff. spp.) that have very specific adaptations to distinct plant hosts.

*Blumeria graminis* f. sp. *hordei* is barley adapted and in this compatible interaction the plants develop powdery mildew colonies on the leaf surface. The fungus penetrates through the cell wall of epidermal cells and produces a haustorium, which is the fungal feeding structure inside the plant cell that obtains nutrients from the plant. This enables the fungus to proliferate rapidly on the surface of the leaf and produce epiphytic mycelium and additional secondary haustoria. Approximately 5 days after inoculation, the fungal colony is visible to the naked eye, and subsequently the colony begins to produce conidiophores, which generate a large number of conidia (asexual spores)^6^. These are airborne and can distribute the fungus to other host plants that can be kilometers away. The yield losses from infected barley can be up to 20%^7^.

The nonhost interaction can be observed between barley and other ff. spp. of *B. graminis* such as *B. graminis* f. sp. *tritici* adapted to wheat (*Triticum aestivum* L.)^8^ or *B. graminis* f. sp. *avenae*, the powdery mildew of oat (*Avena sativa* L.). In such nonhost interactions, penetration is stopped at the cell walls by formation of papillae and/or HR. It has been reported^9^ and we also observed that a few barley varieties (including var. Golden Promise) permitted development of haustoria, but these were smaller and did not allow nutrients to be transferred from the plant to develop conidiophores. The factors determining that barley is a host to f. sp. *hordei*, but a nonhost to f. sp. *tritici*, have not been identified. PTI and ETI are dynamic processes during which an oxidative burst, hormonal changes, and transcriptional reprogramming are triggered. Thus, signal transduction in plant plays an extremely important role in this process^10^.

Early responses of plants to pathogen detection include Ca^2+^ influx, production of reactive oxygen and nitrogen species (ROS and RNS) and NO signaling, which all act as secondary messengers^11^. Higher production of NO can sometimes be observed (NO burst) upon a pathogen attack, but it seems to be closely dependent on the genetic makeup of the plant and of the pathogen^12^. In resistant plants, ROS and RNS modulate gene expression, induce structural defenses or local cell death. Since the HR is accompanied by the generation of excess amounts of oxidative molecules, the fate of cells surrounding the infection site is determined by their ability to tolerate this oxidative stress^13,14^. NO can be removed from cell compartments in many ways, but a special role is attributed to plant phytoglobins (previously known as non-symbiotic hemoglobins)^15^. There are three phylogenetic classes of phytoglobins in plants: classes 1, 2 and 3, of which the structure of classes 1 and 2 more closely resembles that of the human and animal hemoglobins, whereas class 3 resemble that of truncated hemoglobins from prokaryotes. Removal of NO is a well‐documented function of class 1 phytoglobins and the overexpression of such a phytoglobin leads to lower NO production both in Arabidopsis^15^ and in barley^16^. However, the role of NO and plant phytoglobins during pathogen nonhost response is not understood.

In this study, we investigated the responses of barley to a non-adapted isolate of the *B. graminis* f. sp. *tritici*. Barley plants of the cultivar Golden Promise without (wild type) and with overexpression of a phytoglobin (previously hemoglobin) class 1 gene were used17. Most of the current research on nonhost response has focused on gene expression. We show that the gap in knowledge can be bridged by plant proteome responses using quantitative mass spectrometry analysis (LC-MS/MS), which is a powerful tool for identification and quantification of proteins in complex biological samples, allowing us to confirm observed physiological changes and explain them at the molecular level.

The objectives of this study were (i) to identify specific proteins or protein groups that are significantly changed in the wild type during nonhost response to *B. graminis* f. sp. *tritici*, (ii) to examine differences in the leaf proteome during nonhost responses between the wild type and phytoglobin-overexpression plants and (iii) to broaden the understanding of the mechanisms responsible for nonhost response including the role of phytoglobin.

## Results

### Proteomic overview

A total of 8804 protein were identified and quantified in genotype/treatment combinations (WT, HO, WTi and HOi) by LC-MS/MS analysis (Supplementary Table S1). Proteins with q-value <0.05 in LimmaRP analysis^18^ and detected in at least two biological replicates, were regarded as differentially abundant proteins (DAP). Based on these criteria, 1044 DAP were identified. The DAP were analyzed to show the genetic factor – overexpression of phytoglobins (comparison of protein abundance between HO and WT samples and between HOi and WTi samples) and nonhost resistance (comparison of protein abundance between WTi and WT samples and between HOi and HO samples) (Table 1). The number of DAP was highest for inoculated WT (WTi/WT comparison) with 431 proteins, which was almost twice as many as for inoculated HO (HOi/HO) with 220 proteins. The lowest number of DAP (144) was observed for the genotype comparison HO/WT.

**Table 1:** Distribution of differentially accumulated proteins (DAP) in comparisons between barley leaves of genotypes and between barley leaves of uninoculated/inoculated (with *Blumeria graminis* f. sp. *tritici*) plants.

Description	Protein number
DAP between HO and WT	144
DAP between HOi and WTi	249
DAP between WTi and WT	431
DAP between HOi and HO	220

WT, wild-type; HO, phytoglobin overexpressor; WTi, wild-type inoculated; HOi, phytoglobin overexpressor inoculated.

The largest differences in proteins abundance between the treatments after sparse partial-least-squares discriminant analysis (sPLS-DA) are shown by component 1 (4.9%) that explains the variance between inoculated and mock treatment and component 2 (3.5%), that demonstrates difference between genotypes (Supplementary Fig. S1A). The loading values from the analysis are shown in Supplementary Table S2. The 10 proteins that showed the largest change in abundance in the inoculated compared to uninoculated plants are shown in Supplementary Fig. S1B. Among those proteins were heat shock 70 kDa protein (BAJ88744.1), catalases (BAJ93193.1; AAA96948.1; AAC17730.1), and disulfide isomerases (AAA70345.1; BAJ84858.1; AAA70346.1). Scavenging of reactive oxygen species (ROS) play an important part in the response of plants to fungal infection^18^, and catalases remove H_2_O_2_ as part of the plant’s antioxidant systems. Disulfate isomerases catalyzes the breaking and formation of disulfide bonds between cysteine residues and their functions are related to redox regulation and protein folding^19^. This shows how important the redox balance is for the progression of the fungal infection.

#### Genotype differences

**Figure 1:**
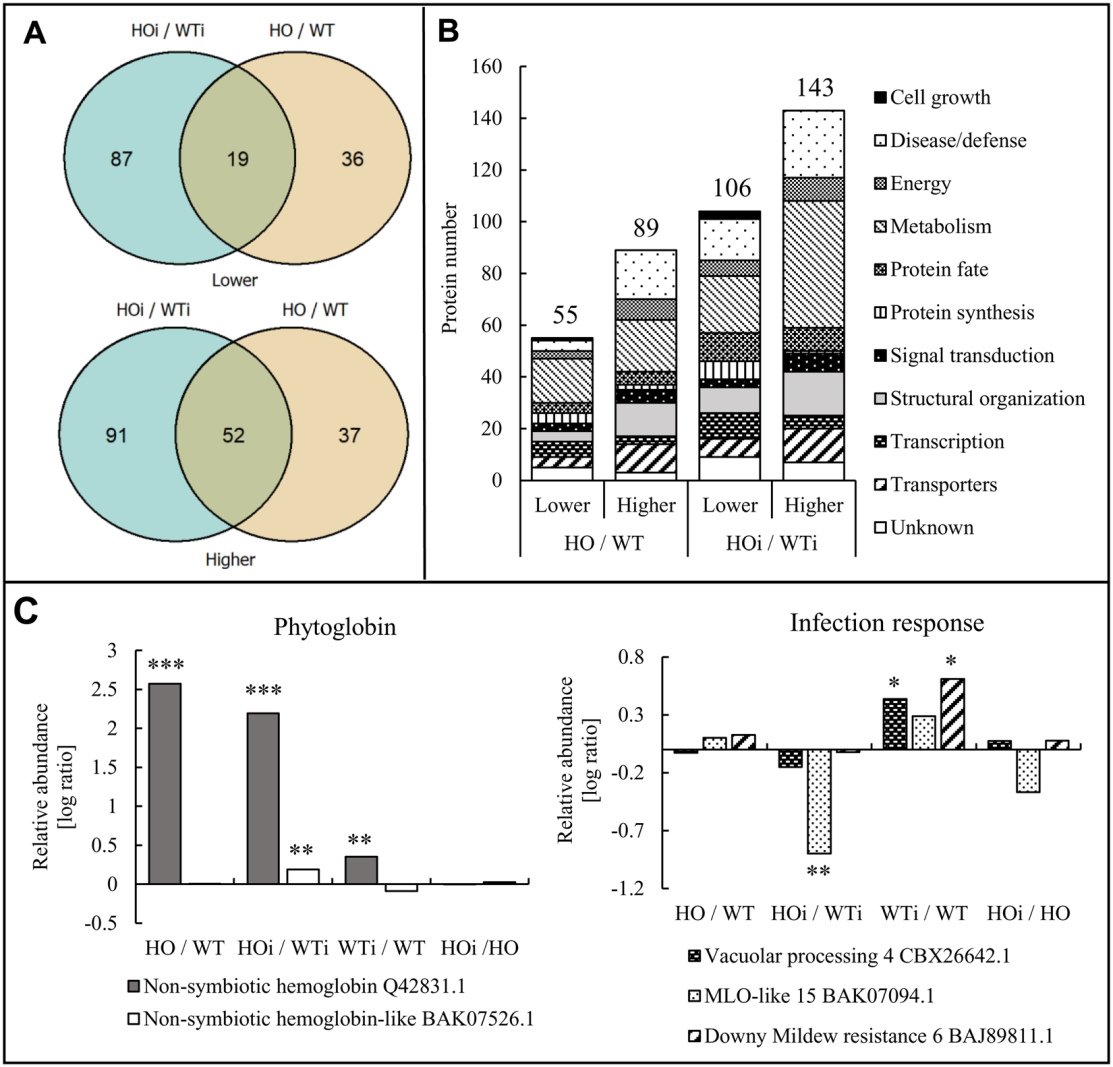
Genotype differences in the leaf proteome. (**A**) Comparison of the number of DAP that were present at lower and higher abundance in HOi/WTi and HO/WT seedlings and the overlap in protein identity between them. (**B**) The functional distribution of the DAP proteins in HOi/WTi and HO/WT seedlings whose abundance was decreased (lower) or increased (higher). (**C**) The content of phytoglobin proteins in all comparisons between treatments and selected examples of proteins associated with the response to pathogen attack. The asterisk indicates significant q-values (‘*’ – q < 0.05, ‘**’ – q < 0.01, ‘***’ – q < 0.001).

We found 106 DAP that decreased in abundance in HOi compared to WTi (HOi/WTi) and only 55 DAP in HO/WT (Fig. 1A). Of these, 19 DAP were the same before and after fungal inoculation. A larger number of DAP became more abundant in HO both after inoculation (143 for HOi/WTi) and before (89 for HO/WT). Here the overlap was 52 DAP (Fig. 1A).

The identified DAP in each comparison were assigned to 11 functional categories using the UniProt database according to category list in Bevan *et al*.^21^. The main functional categories were cell growth, disease/defense, energy, metabolism, protein fate, protein synthesis, signal transduction, structural organization, transcription, transporters and unknown. Each of the categories had several subcategories (Supplementary Table S3). The DAP share in each category differed between the individual comparisons (Fig. 1B). The highest number of DAP for HO/WT and HOi/WTi comparisons were in the metabolism category (37 for HO/WT and 71 for HOi/WTi). For both of these comparisons important groups also included disease/defense (23 for HO/WT and 42 for HOi/WTi), structural organization (17 for HO/WT and 27 for HOi/WTi) and transporters (13 for HO/WT and 15 for HOi/WTi) (Fig. 1B).

Hemoglobin-overexpressing barley plants were previously shown to have a 50-70 fold increase in hemoglobin mRNA compared to WT^17^. Using quantitative proteomics, we here confirmed a higher abundance of nonsymbiotic hemoglobin protein (currently called phytoglobin, Q42831.1) in the overexpressing plants compared to WT, both before and after fungal inoculation (Fig. 1C). A small, but significant, increase in the abundance of this protein was also observed in WTi/WT. A small increase in the abundance of another class 1 phytoglobin (BAK07526.1) was noted as well, but only in HOi/WTi (Fig. 1C). Vacuolar processing enzyme 4 (VPE, CBX26642.1) is a vacuole-localized cysteine proteinase responsible for the maturation and activation of vacuolar proteins, but it also exhibit caspase-1-like activity and that, by controlling vacuolar rupture, is essential for programmed cell death (PCD)^22^. VPE had higher abundance only in the WTi/WT comparison. In the same comparison, Downy mildew resistance protein 6 (BAJ89811.1), which is locally induced in response to pathogen attack^23^, also had a higher abundance. Likewise, the MLO-like 15 protein (BAK07094.1), which is involved in modulation of pathogen defense and leaf PCD^24^, was present at much lower abundance in HOi compared to WTi (Fig. 1C). Higher abundance of both proteins in WTi/WT could mean that WT plants had a PCD incidence. However, the protein MLO-LIKE 15 (BAK07094.1) was downregulated in HOi/WTi. This protein dampens the cell wall-restricted hydrogen peroxide burst at points of attempted fungal penetration of the epidermal cell wall, and in subtending mesophyll cells, so it suppresses a second oxidative burst and PCD^24^. Therefore, plants characterized by a reduced amount of MLO should have an increased frequency of PCD. The results obtained by Sørensen *et al*.^8^ indicate that this is the case in plants with overexpression of phytoglobins during nonhost response.

#### Differences caused by fungal infection

**Figure 2:**
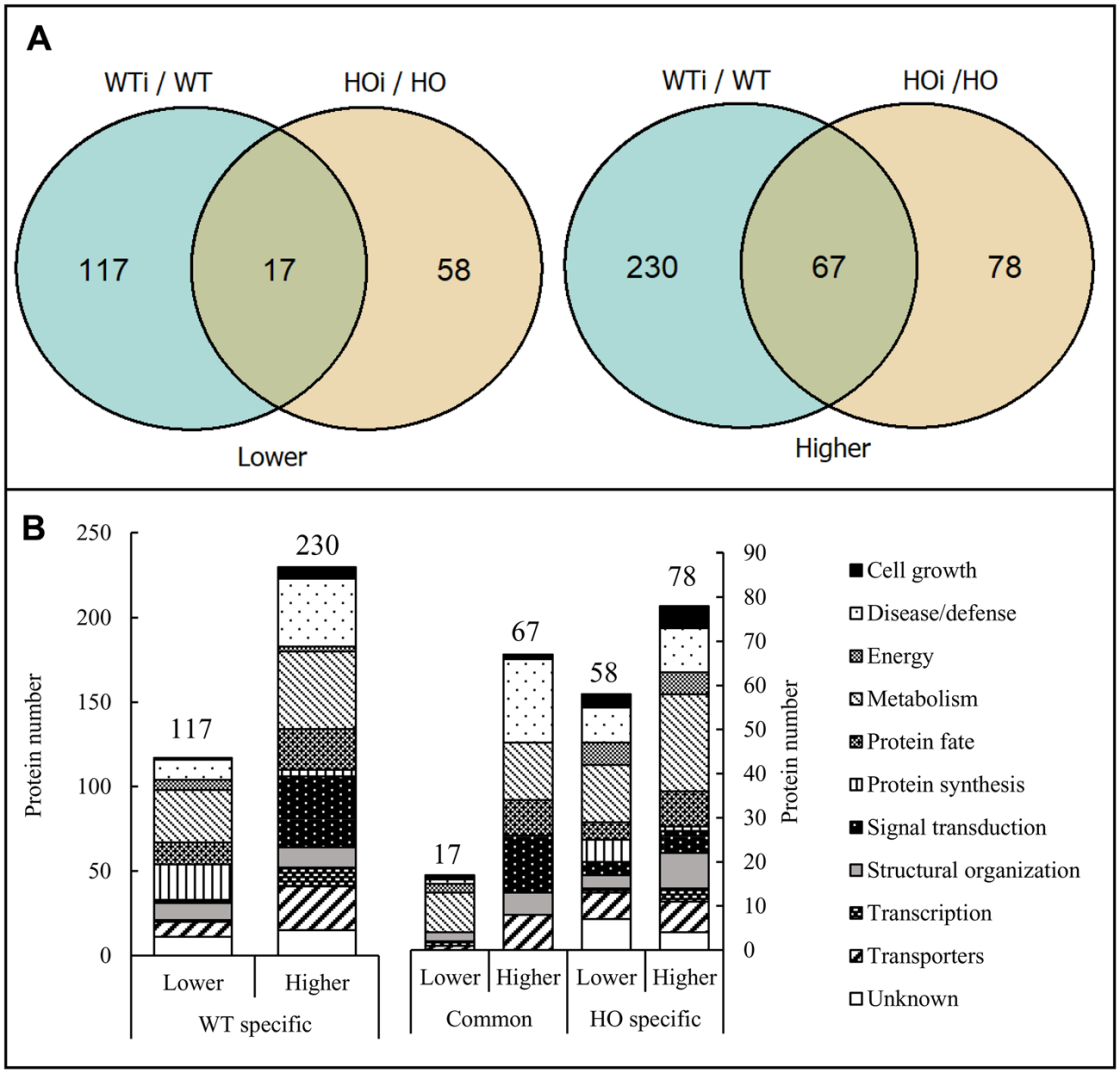
Fungal infection differences induced in the barley leaf proteome. (**A**) Comparison of the number of DAP that were accumulated higher and lower abundance in seedlings after inoculation depending on their genotype (WT – wild type, HO – overexpression of phytoglobin), and the overlap in protein identity between them. (B) The functional distribution of proteins that were specific for wild type (WTi), overexpressed plants (HOi) after inoculation and the DAP they had in common.

We found 134 DAP that were lower in abundance after inoculation in WT (WTi/WT) and only 75 DAP in HO (HOi/HO) (Fig. 2A). Of these, 17 DAP were common for the two comparisons. Many more DAP were observed to have increased abundance after inoculation – 297 in WT (WTi/WT) and 145 in HO (HOi/HO). Of these, 67 DAP were common for the two comparisons (Fig. 2A).

The DAP identified as a result of inoculation were divided into those specific for WT or HO and those identical for the two genotypes (Fig. 2B). The distribution of the number of DAP belonging to functional categories differed markedly between WT and OH. The highest number of DAP were related to metabolism (77 in WT, 35 in HO, 22 in common), disease/defense (52 in WT, 18 in HO, 20 in common), as well as signal transduction (44 in WT, 8 in HO, 13 in common) with the exception of HO specific where the 3rd largest group were proteins associated with transporters (13 DAP)(Fig. 2B). This is consistent with the observation that during a pathogen attack, the plant activates a network of pathways to resist the pathogen invasion, which usually requires thousands of protein^10^. WT plants were characterized by twice as many DAP proteins than the plants with overexpression of phytoglobin (Table 1), that confirms what was found by Sørensen *et al*.^8^ in other words that overexpression of Hb suppresses the response to pathogens.

In the following sections we will describe DAP belonging to specific functional categories and sub-categories particularly relevant to the inoculation process, the innate immunity of the two genotypes and the signal transduction pathways. Time point selected for analysis was 72 hai as the progression of inoculation is different between two genotypes as determined by Sørensen *et al*.^8^. Barley plants with overexpression of hemoglobin were characterized by less papillae formation and higher rate of hypersensitive reaction both with and without haustorium formation after 72 hai. This data published by Sørensen *et al*.^8^ is summarized in Supplementary Table S4.

### DAP related to photosynthesis and chlorophyll metabolism

**Figure 3:**
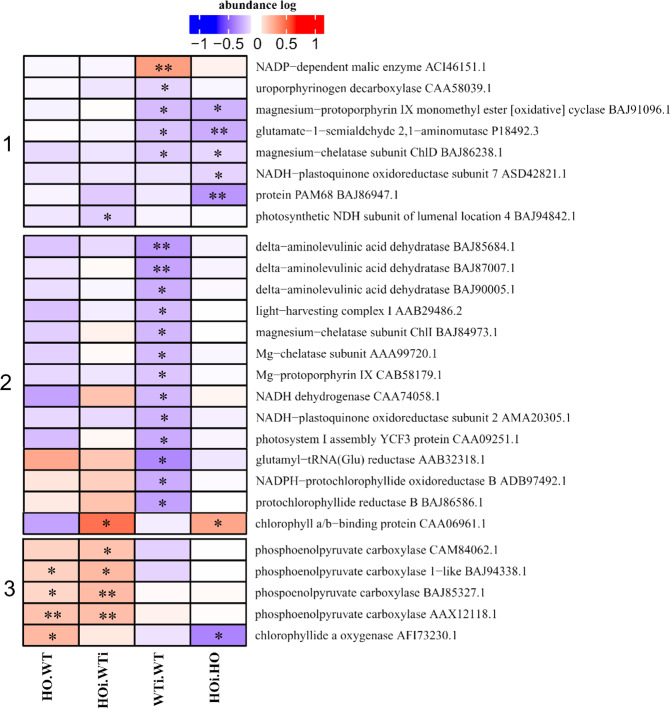
Heatmap displaying the comparison of abundance of DAP with function related to photosynthesis and chlorophyll metabolism. Seedlings of wild type shown as WT and with overexpression of phytoglobin as HO, seedlings after inoculation shown as WTi (wild type) and HOi (overexpressed phytoglobin). The color scale illustrates the average relative abundance level of each protein for the 3 biological samples; red and blue indicate higher and lower abundance for each comparison, respectively. The color intensity indicates the degree of protein up- or downregulation. The asterisk indicates the q-value of significant values (‘*’ – q < 0.05, ‘**’ – q < 0.01, ‘***’ – q < 0.001).

Sørensen *et al*.^8^ showed that the leaf chlorophyll content in WT plants decreased more than in HO plants after compatible infection by *B. graminis* f. sp *hordei* (A6 isolate). We therefore focused on the DAP proteins associated with the metabolism of chlorophyll. The DAP belonging to the subcategories of chlorophyll (metabolism function) and photosynthesis (energy function) were analyzed together (Fig. 3) and fell into three clusters. In cluster 1 are proteins that decreased in abundance in both WTi/WT and HOi/HO or only in HOi/HO. In cluster 2 are DAP the abundance of which decreased in WTi/WT and in the cluster 3 are DAP that increased in HO/WT and HOi/WTi (Fig. 3). The observed decrease in abundance for DAP involved in chlorophyll biosynthesis is consistent with the observation that WTi plants had a significantly lower chlorophyll content than HOi plants in the compatible interaction as early as 3 days after infection^8^. A decreased reduction in the transcription of genes encoding proteins involved in chlorophyll biosynthesis has also been observed in nonhost response of barley to other fungal pathogens^25^. This may indicate a change in the type of response to an attack and change in energy status in plants with overexpression of phytoglobin connected to better photosynthesis efficiency.

### DAP connected with protein synthesis

**Figure 4:**
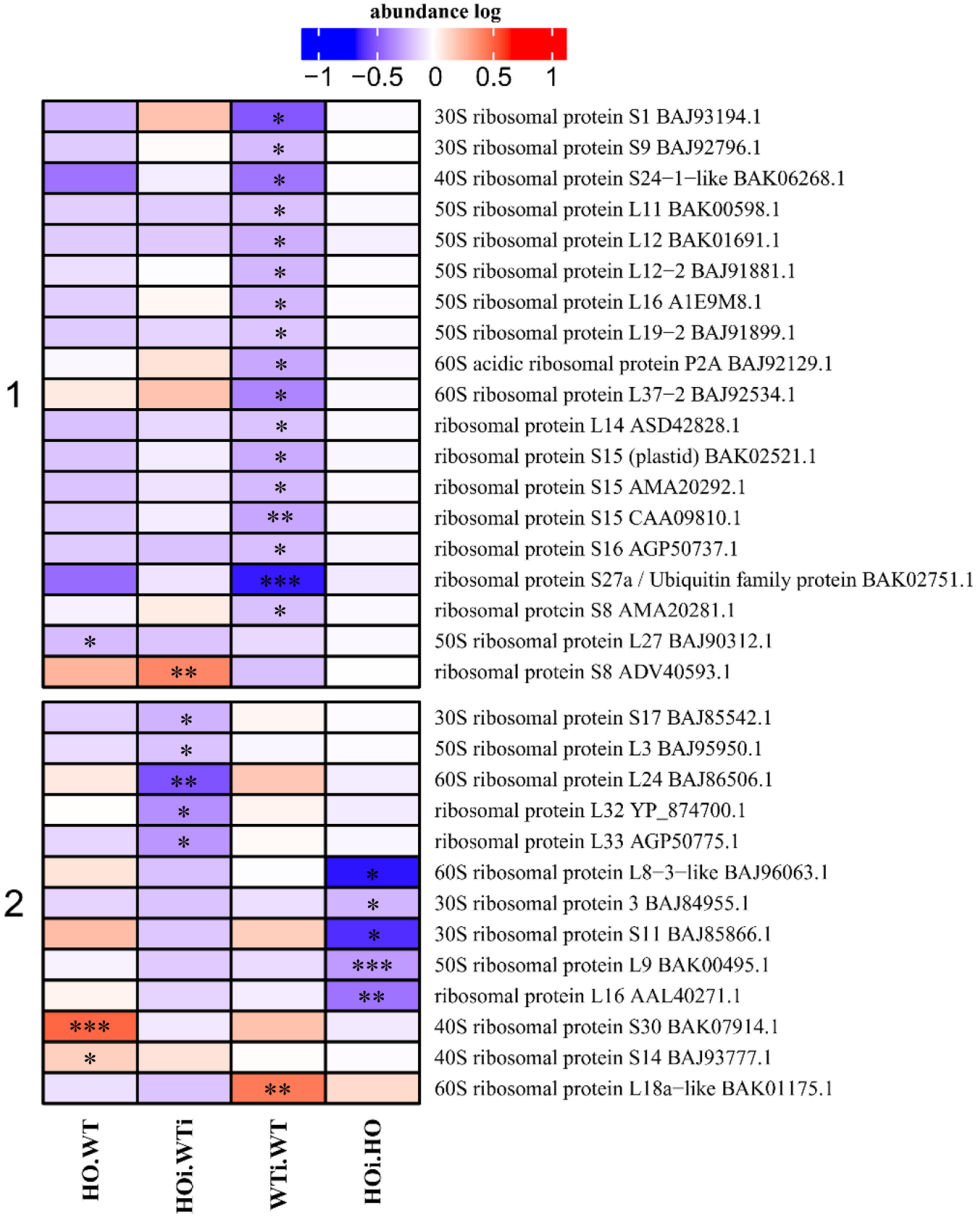
Heatmap displaying the comparison of abundance of DAP with function related to protein synthesis. Seedlings of wild type shown as WT and with overexpression of phytoglobin as HO, seedlings after inoculation shown as WTi (wild type) and HOi (overexpressed phytoglobin). The color scale illustrates the average relative abundance level of each protein for the 3 biological samples; red and blue indicate higher and lower abundance for each comparison, respectively. The color intensity indicates the degree of protein up- or downregulation. The asterisk indicates the q-value of significant values (‘*’ – q < 0.05, ‘**’ – q < 0.01, ‘***’ – q < 0.001).

One of the early plant responses to the pathogen attack is likely to be connected with changes in protein synthesis. Among the DAP belonging to the protein synthesis category, three subcategories were identified - tRNA splicing, translational factors and ribosomal protein. The largest DAP group among these subcategories were ribosomal proteins (Fig. 4), and the ribosomal DAP could be divided into 2 clusters. Cluster 1 comprised DAP which almost all decreased in abundance in WTi/WT (Fig. 4). Cluster 2 comprised 5 DAP with decreased abundance in HOi/WTi and 5 DAP with lower abundance in HOi/HO. There were also two DAP that increased in abundance in HO/WT and one DAP that increased in WTi/WT (Fig. 4).

The observed decrease in the abundance of ribosomal proteins in WTi indicates that protein synthesis was less active after infection in WT. However, ribosomal proteins may also play a more direct role in defense against pathogens. *Nicotiana benthamiana* plants where the genes encoding ribosomal proteins L12 and L19 had been silenced showed varying extent of delay in initiation of HR against nonhost pathogens *X. campestris* pv. vesicatoria and *P. syringae* pv. tomato T1^26^.

### Disease/defense-related proteins

Successful plant defense depends on an early and rapid perception of the invading pathogen and subsequent induction and mobilization of biochemical and structural defense-related mechanisms. When comparing protein abundance within each genotype, we wanted to know which of the proteins associated with disease/defense response were changed in response to inoculation and if barley activates fundamentally different responses depending on the expression of phytoglobins.

**Figure 5:**
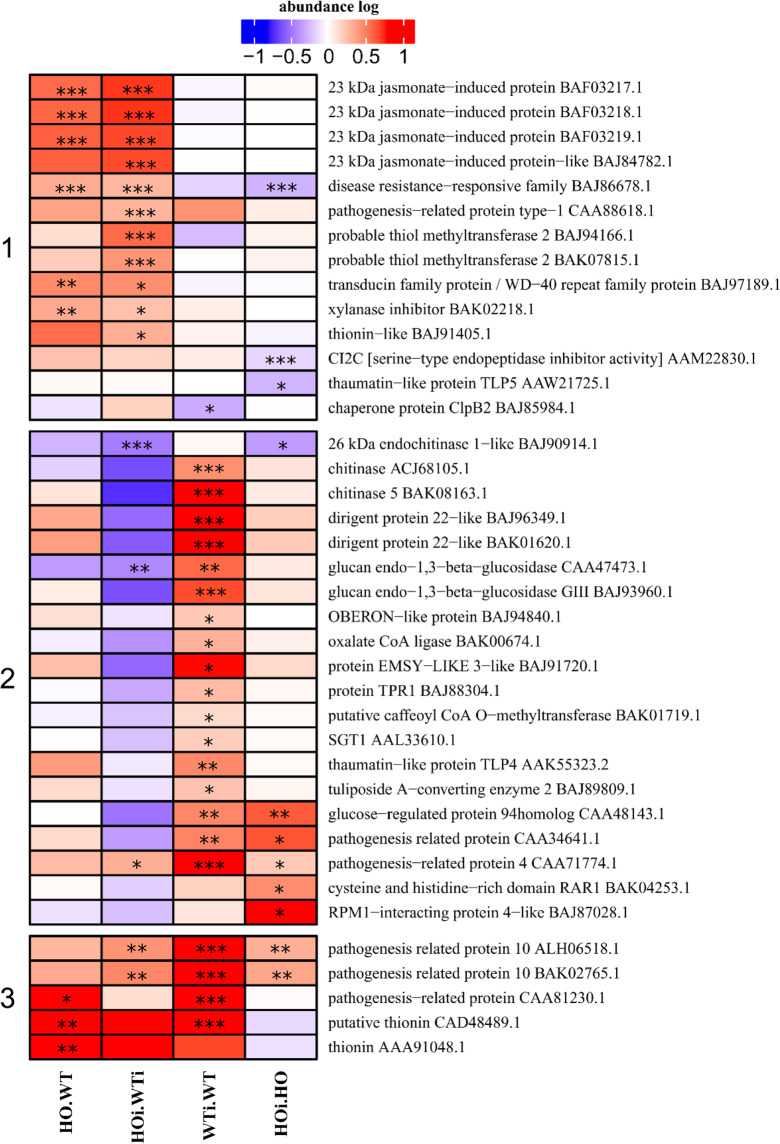
Heatmap displaying the comparison of abundance of DAP related to defense function. Seedlings of wild type shown as WT and with overexpression of phytoglobin as HO, seedlings after inoculation shown as WTi (wild type) and HOi (overexpressed phytoglobin). The color scale illustrates the average relative abundance level of each protein for the 3 biological samples; red and blue indicate higher and lower abundance for each comparison, respectively. The color intensity indicates the degree of protein up- or downregulation. The asterisk indicates the q-value of significant values (‘*’ – q < 0.05, ‘**’ – q < 0.01, ‘***’ – q < 0.001).

**Figure 6:**
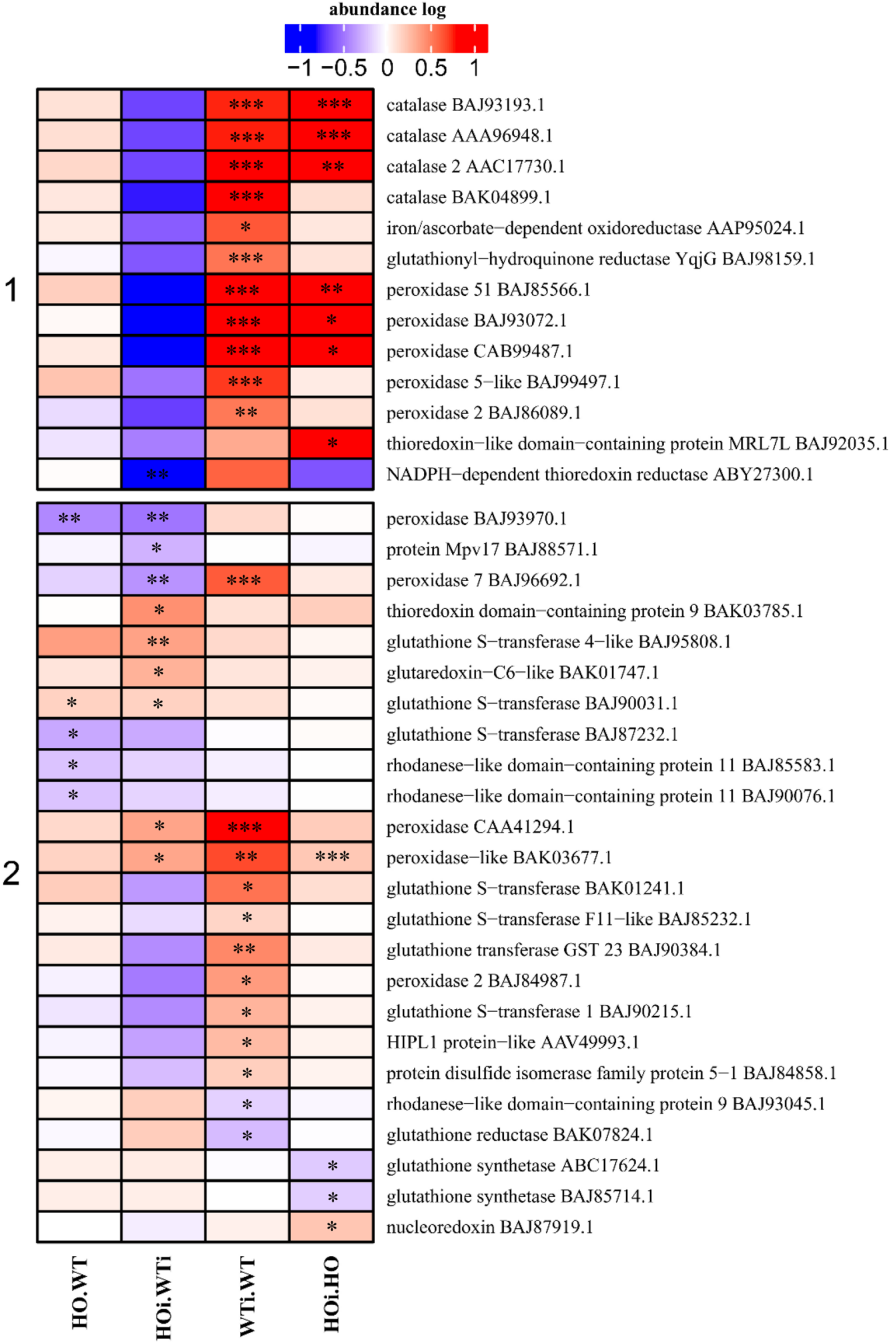
Heatmap displaying the comparison of abundance of DAP related to ROS metabolism. Seedlings of wild type shown as WT and with overexpression of phytoglobin as HO, seedlings after inoculation shown as WTi (wild type) and HOi (overexpressed phytoglobin). The color scale illustrates the average relative abundance level of each protein for the 3 biological samples; red and blue indicate higher and lower abundance for each comparison, respectively. The color intensity indicates the degree of protein up- or downregulation. The asterisk indicates the q-value of significant values (‘*’ – q < 0.05, ‘**’ – q < 0.01, ‘***’ – q < 0.001).

In the category of disease/defense the DAP were assigned to one of three subcategories – defense-related (Fig. 5), ROS metabolism (Fig. 6) and stress response. Defense-related DAP were separated into 3 clusters (Fig. 5). In cluster 1 are proteins that increased in abundance in the HOi/WTi comparison or in both HOi/WTi and HO/WT. The exceptions were four proteins of which two (CI2C - AAM22830.1; thaumatin-like protein TLP5 - AAW21725.1) had a reduced abundance in HOi/HO. While chaperone protein CLpB2 (BAJ85984.1) had a lower abundance in WTi/WT and disease resistance-responsive family (BAJ86678.1) was more abundant in HOi/WTi and HO/WT and less abundant in HOi/HO. Cluster 2 comprises mainly DAP which increased in abundance in WTi/WT or in HOi/HO or both. Three other proteins were also included in this cluster. For the first of them, pathogenesis related protein 4 (CAA71774.1), the abundance increased in 3 comparisons (WTi/WT, HOi/HO and HOi/WTi). The second, glucan endo-1,3-beta-glucosidase (CAA47473.1), had increased abundance in WTi/WT, and decreased in HOi/WTi. The last protein in this cluster was 26 kDa endochitinase 1-like (BAJ90914.1) the abundance of which decreased in both HOi/WT and in HOi/HO. Cluster 3 comprises only 5 DAP and they are characterized by higher abundance in HOi/WTi, WTi/WT and HOi/HO or in HO/WT and WTi/WT (Fig. 5).

WT plants were characterized by a larger number of upregulated DAP associated with the defense function. One of these was the SGT1 protein, which is part of the SCF complex (Skp1–Cullin–F-box protein) that targets regulatory proteins for degradation and is essential for some of the *R* gene-mediated disease resistances. It was shown that silencing of SGT1 is causing break in the resistance of *N. bentamiana* to some of nonhost pathogens^27^. The exact role of *SGT1* in barley resistance to *B. graminis* f. sp. *tritici* has yet to be determined. Among other proteins, two chitinases (ACJ68105.1 and BAK08163.1) were among the DAP that increased in abundance in WTi/WT (Fig. 5). But the third, 26 kDa endochitinase was downregulated in both HOi/HO and HOi/WTi (Fig. 5), which may also be connected with different responses of these two genotypes to a pathogen attack. Barley papillae formed in incompatible interactions have significantly higher accumulation of thionin proteins^28^ and ROS^29^ as compared with papillae formed in compatible interactions. Among DAP in our study that were defense related was also putative thionin (CAD48489.1) which increased in abundance in WTi/WT, but not in HOi/HO (Fig. 5).

In a wide range of plant–pathogen interactions bi-phasic bursts of ROS by plants have been observed with a first phase peaking after 20 min and a second phase occurring 4 to 6 h later which has been correlated with plant resistance. The ROS accumulation is regulated by intricate system of scavenging both by enzymes and nonenzymatic ways^30^. Proteins connected with this system were in the detoxification-related (or ROS removal) sub-category that comprises DAP such as catalases, peroxidases, thioredoxin and rhodanese domain containing and proteins connected with glutathione metabolism in two clusters (Fig. 6). In cluster 1 are mainly DAP that increased in abundance in WTi/WT or in WTi/WT and HOi/HO. In this cluster was also a NADPH-dependent thioredoxin reductase (BY27300.1) which decreased in abundance in HOi/WTi. Cluster 2 was more diverse than cluster 1, but most DAP increased in abundance in WTi/WT, or changed abundance (increase or decrease) in HOi/WTi or decreased in abundance in HO/WT. In this cluster was also peroxidase-like (BAK03677.1) which increased in abundance in three comparisons (HOi/WTi, WTi/WT and HOi/HO) (Fig. 6).

The changes observed in abundance of proteins connected with ROS removal are consistent with the observation Sørensen *et al*.^8^ where plants with overexpression of phytoglobin showed lower H_2_O_2_ content and higher peroxidase activity than WT plants.

### DAP related to function connected with structural organization of the cell wall

**Figure 7:**
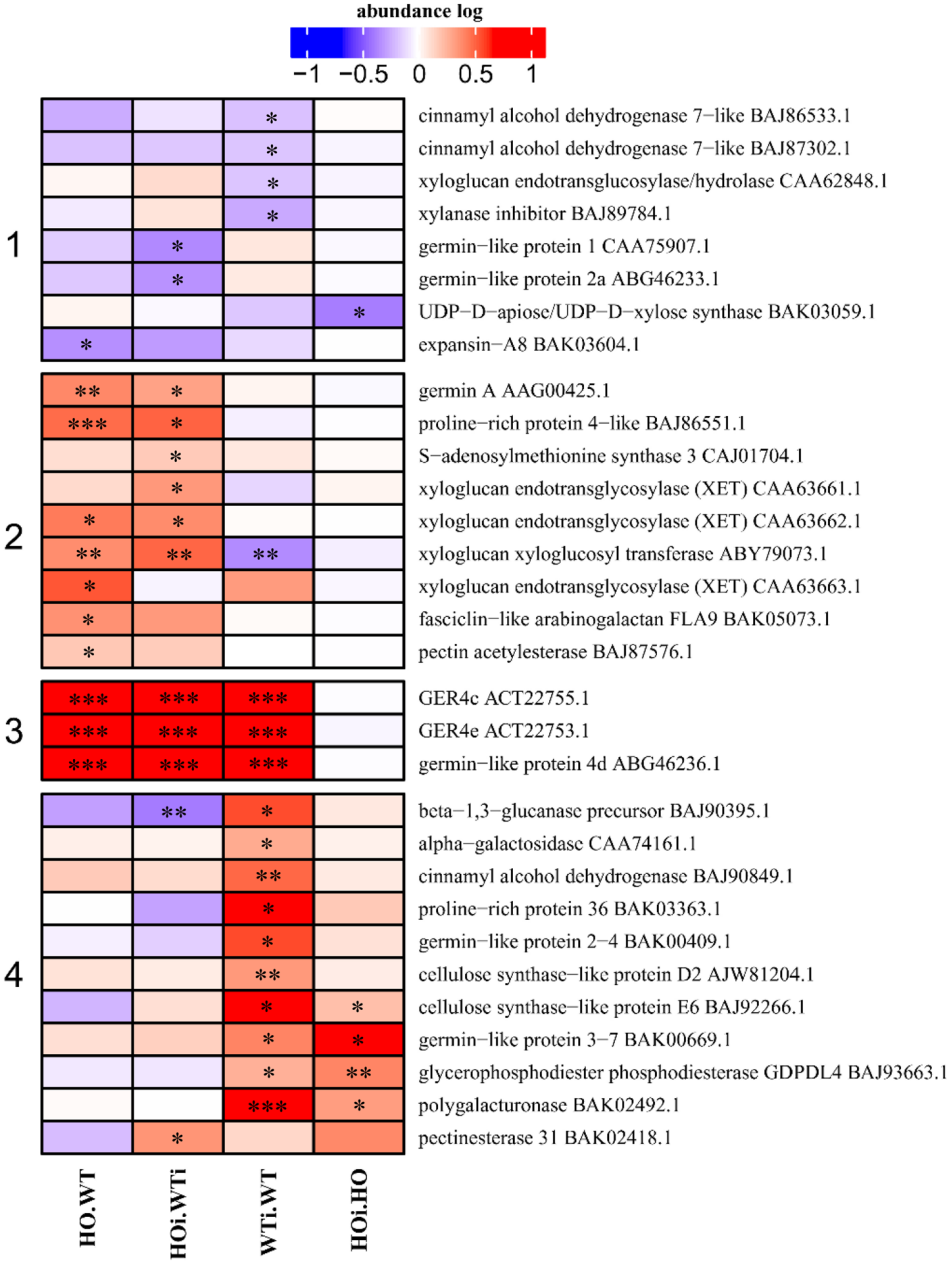
Heatmap displaying the comparison of abundance of DAP with function related to the cell wall. Seedlings of wild type shown as WT and with overexpression of phytoglobin as HO, seedlings after inoculation shown as WTi (wild type) and HOi (overexpressed phytoglobin). The color scale illustrates the average relative abundance level of each protein for the 3 biological samples; red and blue indicate higher and lower abundance for each comparison, respectively. The color intensity indicates the degree of protein up- or downregulation. The asterisk indicates the q-value of significant values (‘*’ – q < 0.05, ‘**’ – q < 0.01, ‘***’ – q < 0.001).

**Figure 8:**
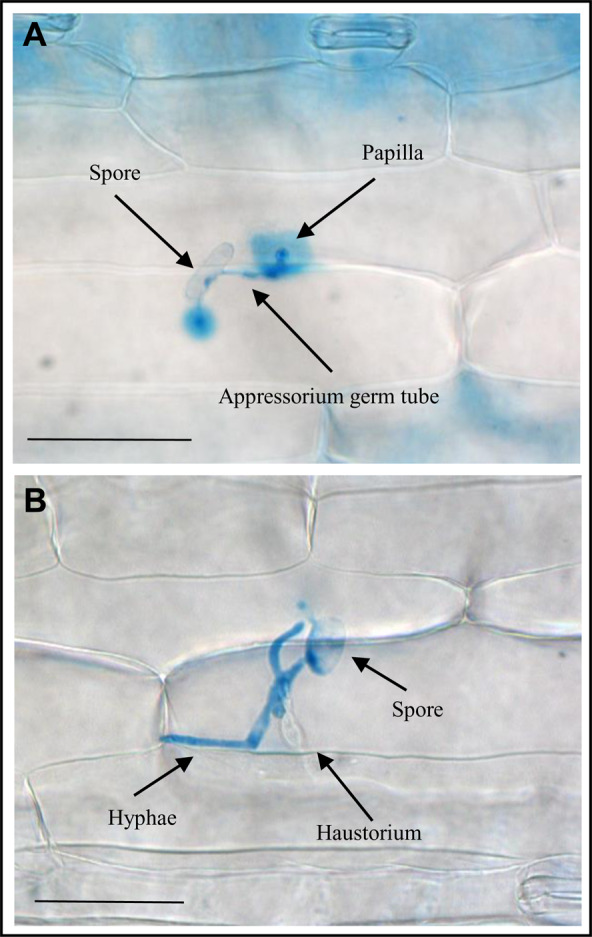
Microscopic pictures of fungi in the nonhost response of Golden Promise barley seedlings inoculated with isolates of *Blumeria graminis* adapted to wheat taken after 72 hai. (A) Papillae formation and (B) haustorium formation. All scale bars = 50 μm.

Compared with many plant defense responses that can be specific to a phylum or even a species, the formation of callose-rich papillae can be regarded as a ubiquitous response because it appears to be induced in essentially all plants following pathogen challenge and plays a very important role during the nonhost response. In most cases, it is enough to stop the pathogen attack at the preinvasion stage^31^. Furthermore it was shown that papillae formation happens less often in plants with overexpression of phytoglobin, and the infection is more advanced (Supplementary Table S4). Among the subcategories of structural organization we here focused on the DAP connected to organization of cell wall (Fig. 7) as it has the greatest relevance for papillae formation. DAP in this subcategory were enzymes like cellulose synthase, germin-like, xyloglucan endotransglycosylaese or cinnamyl alcohol dehydrogenase and they could be divided into 4 clusters. In cluster 1 were DAP for which the abundance decreased in one of the comparisons. In cluster 2 where mainly DAP that increased in abundance in HO/WT or HOi/WTi or both. Cluster 3 was uniform with three germin proteins that showed higher abundance in three comparisons - HO/WT, HOi/WTi and WTi/WT. In cluster 4 where mainly DAP that increased in abundance in WTi/WT or both WTi/WT and HOi/HO. The exception were two DAP - beta-1,3-glucanase precursor (BAJ90395.1) that had higher abundance in WTi/WT and lower abundance in HOi/WTi and pectinesterase 31 (BAK02418.1) that showed higher abundance only in HOi/WTi (Fig. 7). We observed three GER4 proteins increased in abundance in HO/WT and their level was still higher in HOi/WTi. There was no significant difference between HO before and after inoculation. However, their abundance increased in WT plants after inoculation (Fig. 7, cluster 3). This suggests that NO is taking part in regulation of germin-like proteins that have superoxide dismutase activity and function in PAMP-triggered immunity^32^. Most of the cell wall proteins had an elevated abundance only in WT plants after inoculation (Fig. 7, cluster 4), including cinnamyl alcohol dehydrogenase, cellulose synthase-like, alpha-galactosidase or beta-1,3-glucanase precursor. It was observed previously^8^ that overexpression of phytoglobins in barley plants led to changes in response to nonadapted *B. graminis* (H8). HO plants had less papilla formation and thus partial formation of haustoria was observed after 72 hai (Fig. 8).

### DAP related to transporters

**Figure 9:**
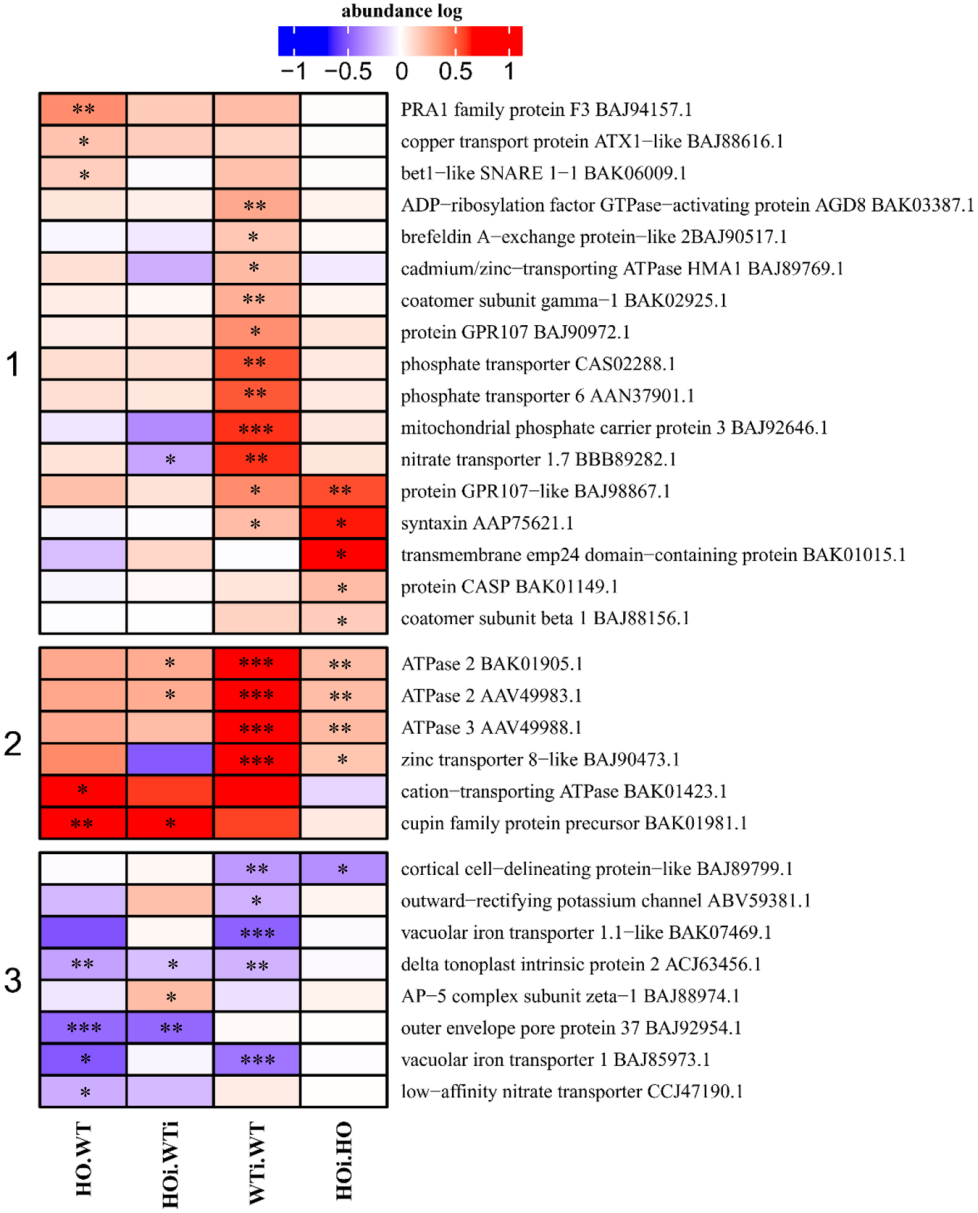
Heatmap displaying the comparison of abundance of DAP with function related to ion and vesicle transport. Seedlings of wild type shown as WT and with overexpression of phytoglobin as HO, seedlings after inoculation shown as WTi (wild type) and HOi (overexpressed phytoglobin). The color scale illustrates the average relative abundance level of each protein for the 3 biological samples; red and blue indicate higher and lower abundance for each comparison, respectively. The color intensity indicates the degree of protein up- or downregulation. The asterisk indicates the q-value of significant values (‘*’ – q < 0.05, ‘**’ – q < 0.01, ‘***’ – q < 0.001).

During papilla deposition, site-directed transport of papilla components, cell wall-synthesizing enzymes, and other components is needed. Thus, an induction and regulation of cell proteins related to transportation and vesicle trafficking would be expected. DAP in the functional category transporters were divided into nine subcategories (Supplementary Data Table S3). DAP belonging to three of these subcategories – ions, nitrate and vesicles - were divided into 3 clusters (Fig. 9). In cluster 1 most of the DAP increased in abundance in WTi/WT, but three proteins increased in abundance in HO/WT and a few increased in abundance in HOi/HO. Cluster 2 comprises DAP that increased in abundance in two or three comparisons with the exception of cation-transporting ATPase (BAK01423.1) that increased only in HO/WT. Cluster 3 contains the DAP that decreased in abundance in one or several of the comparisons (Fig. 9).

During the nonhost response of barley to *B. graminis* f. sp. *tritici* modulation of plant H^+^-ATPases abundance was observed (Fig. 9). Three ATPases were upregulated in both WTi/WT and HOi/HO, but in WT plants slightly higher abundance was observed. One Cd^2+^/Zn^2+^-transporting ATPase was only upregulated in WT plants after inoculation. H^+^-ATPases are the major pumps in charge of transporting ions thru plasma membrane^33^. Furthermore, plasma membrane potential can be regulated by activation or inhibition of the ATPase, in that way it influence other transporters and control ion flux^34^. Various pathogens PAMPs can modify transport of ions and cause alkalization of cytoplasm, that why plant first cellular response is connected with modification of extracellular pH^35^.

Proteins connected with vesicle trafficking were upregulated in HOi/HO, e.g. protein GPR107-like (BAJ98867.1), transmembrane emp24 domain-containing protein (BAK01015.1), protein CASP (BAK01149.1) or co-atomar subunit beta 1 (BAJ88156.1). In addition, syntaxin (AAP75621.1) increased more in HOi/HO (0.587 ± 0.032 log ratio) than in WTi/WT (0.211 ± 0.035 log ratio) (Fig. 9). Plasma membrane syntaxin with SNAP33 (soluble N-ethylmaleimide-sensitive factor adaptor protein 33) and VAMP721/VAMP722 (vesicle-associated membrane proteins) form SNARE complex that may aid fusion of vesicles with the plasma membrane. Focal accumulation of syntaxin may thereby guarantee that, upon pathogen penetration, the vesicle cargo is released into the apoplast where required. Moreover, syntaxin and SNAP34 are needed for nonhost and basal penetration resistance to powdery mildew fungi in *A. thaliana* and barley, but not for gene-for-gene resistance^36,37^.

### DAP with function related to secondary metabolism

**Figure 10:**
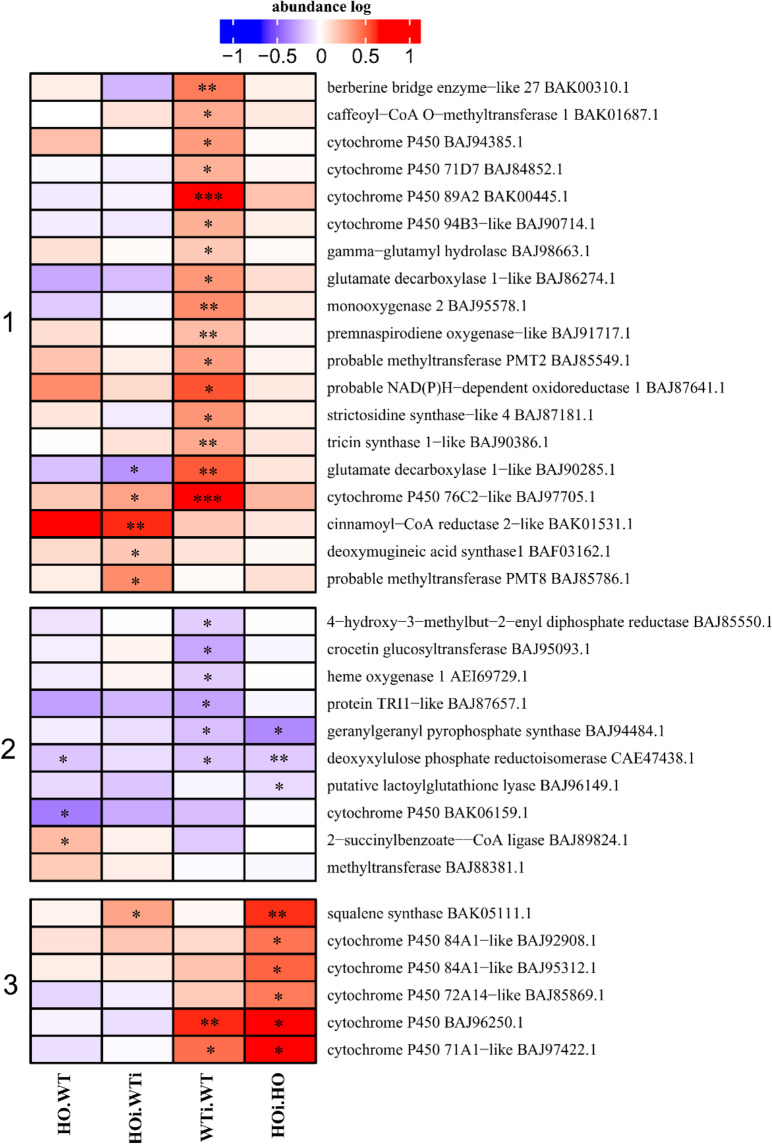
Heatmap displaying the comparison of abundance of DAP with function related to secondary metabolism. Seedlings of wild type shown as WT and with overexpression of phytoglobin as HO, seedlings after inoculation shown as WTi (wild type) and HOi (overexpressed phytoglobin). The color scale illustrates the average relative abundance level of each protein for the 3 biological samples; red and blue indicate higher and lower abundance for each comparison, respectively. The color intensity indicates the degree of protein up- or downregulation. The asterisk indicates the q-value of significant values (‘*’ – q < 0.05, ‘**’ – q < 0.01, ‘***’ – q < 0.001).

During post invasion resistance, the plant cell mounts a defense response through production and secretion of secondary metabolites to the apoplast and initiation of systemic resistance signaling to distant parts of the plant^38^. Among the DAP classified as related to metabolism were also some in the subcategory of secondary metabolism (with three clusters), such as cytochrome P450, berberine bridge enzyme-like, glutamate decarboxylase 1-like or strictosidine synthase-like (Fig. 10). In cluster 1 most DAP were found at a higher abundance in WTi/WT. Three proteins were characterized by higher abundance in HOi/WTi comparison and two were changing in both of these comparisons (glutamate decarboxylase 1-like, BAJ90285.1 and cytochrome P450 76C2, BAJ97705.1). Cluster 2 comprises DAP that decreased in abundance in one of the comparisons – HO/WT, WTi/WT or HOi/HO. Cluster 3 comprises DAP that had higher abundance in HOi/HO or HOi/HO and WTi/WT. The exception was squalene synthase (BAK05111.1) which had a higher abundance in HOi/HO and HOi/WTi (Fig. 10). In general, DAP found in WTi/WT were not found in HOi/HO and vice versa. In other words, the response of the secondary metabolism to infection was strongly affected by hemoglobin/phytoglobin overexpression.

Transport could be also connected to accumulation of secondary metabolites such as phenolics, production of saponins, and production of phytoalexins. Among the DAP proteins involved in secondary metabolism that increased in abundance in response to infection was squalene synthase (BAK05111.1) (Fig. 10, cluster 3). It takes part in terpene and sterol biosynthesis and it was only upregulated in HOi/HO. In few plant species the diterpenes and sesquiterpenes act as phytoalexins, e.g., 14 diterpene phytoalexins have been identified in *Oryza sativa*^39^. Other upregulated proteins in HOi/HO comparisons were five cytochrome P450, two of them were at the same time upregulated in WTi/WT and five others had higher abundance only in WTi/WT (Fig. 10, cluster 1 and 3). Cytochromes P450 participate in a variety of biochemical pathways to produce a vast diversity of plant natural products. Cytochrome P450 genes have been estimated to constitute up to 1% of all the genes in plant genomes, implying that there is a huge variety of cytochrome P450-dependent reactions. Camalexin (3-triazol-2′-ly-indole) is the main phytoalexin of *A. thaliana* and is biosynthesized from indole-3-aldoxime, which is derived from tryptophan by a cytochrome P450-catalyzed reaction^40^. Another enzyme that may take part in barley synthesis of phytoalexin is berberine bridge enzyme-like 27 (BAK00310.1) that had increased abundance in WTi/WT (Fig. 10, cluster 1). Berberine-bridges enzymes are flavin-dependent oxidoreductases that could take part in the biosynthesis of isoquinoline alkaloids. However, their role is not clear in plant families that do not synthesize alkaloids. They are characterized by their exceptionally high upregulation observed during the response to pathogens and contribute to the expressed secretome during infection by various plant pathogens, suggesting a role in plant-pathogen interactions^41^.

### DAP connected to plant signal transduction

**Figure 11:**
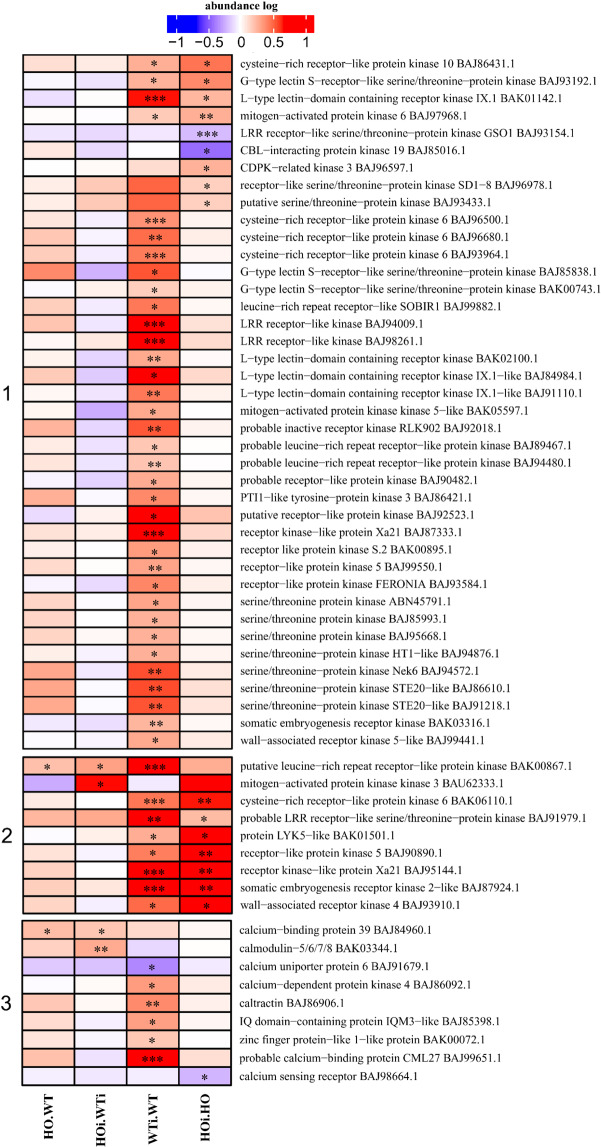
Heatmap displaying the comparison of abundance of DAP related to kinases (1 and 2) and Ca^2+^ signaling (3). Seedlings of wild type shown as WT and with overexpression of phytoglobin as HO, seedlings after inoculation shown as WTi (wild type) and HOi (overexpressed phytoglobin). The color scale illustrates the relative abundance level of each protein across the 3 biological samples; red and blue indicate higher and lower abundance for each comparison, respectively. The color intensity indicates the degree of protein up- or downregulation. The asterisk indicates the q-value of significant values (‘*’ – q < 0.05, ‘**’ – q < 0.01, ‘***’ – q < 0.001).

**Figure 12:**
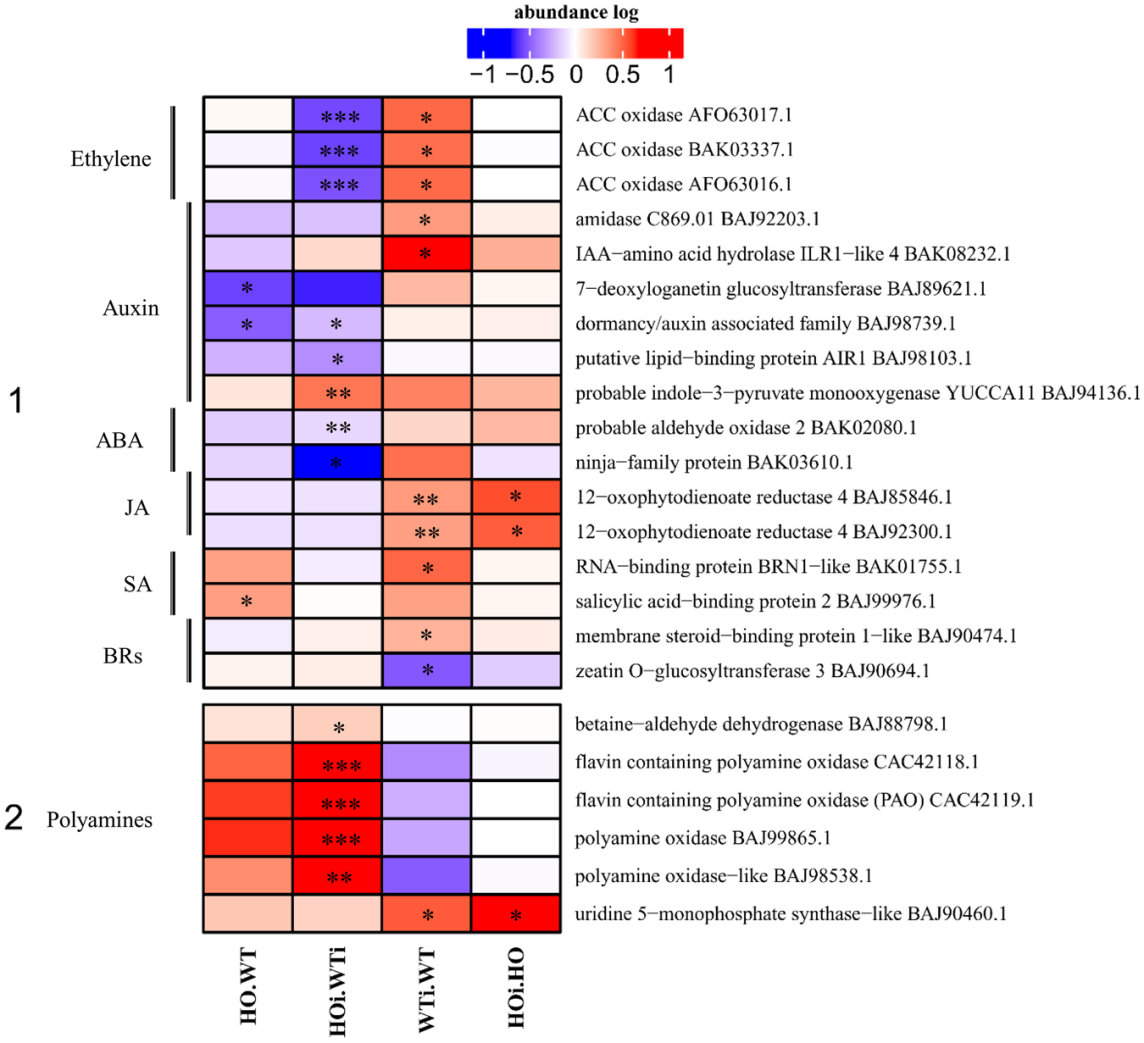
Heatmap displaying the comparison of abundance of DAP related to hormone metabolism and signaling (1) and polyamines metabolism (2). Seedlings of wild type shown as WT and with overexpression of phytoglobin as HO, seedlings after inoculation shown as WTi (wild type) and HOi (overexpressed phytoglobin). The color scale illustrates the average relative abundance level of each protein across the 3 biological samples; red and blue indicate higher and lower abundance for each comparison, respectively. The asterisk indicates the q-value of significant values (‘*’ – q < 0.05, ‘**’ – q < 0.01, ‘***’ – q < 0.001).

NO as signal molecule plays important role in response to environmental changes such as abiotic stress^42,43^ and biotic stress^44^. It also interacts with other signaling molecules such as kinases^45^ and Ca^2+^ ^46^. There were five subcategories in the functional category of signal transduction– hormones, GTPase, kinases, calcium and other. The largest group comprised protein kinases and those taking part in Ca^2+^ signaling, which is why kinases have been divided into two clusters (cluster 1 and 2), and calcium signaling proteins created a cluster 3 (Fig. 11). In cluster 1, most of the kinases increased in abundance in WTi/WT comparisons, but not in HOi/HO. In the same cluster also included three kinases that showed increased abundance in HOi/HO and two that decreased in the same comparison. In cluster 2, the majority of DAP increased in abundance in both WTi/WT and HOi/HO. The exceptions were putative leucine-rich repeat receptor-like kinase (BAK00867.1), which was increased in abundance in three comparisons (HO/WT, HOi/WTi and WTi/WT), and mitogen-activated kinase 3 (BAU62333.1). Cluster 3 comprised calcium signaling DAP and the majority (5) increased in abundance in WTi/WT while one (BAJ91679.1) decreased in abundance in WTi/WT. Two DAP increased in abundance in HOi/WTi, but one of them (calcium-binding protein 39, BAJ84960.1) also increased in abundance in HO/WT. Calcium sensing receptor (BAJ98664.1) abundance was decreased in HOi/HO (Fig. 12). As for secondary metabolism (Fig. 11), a general trend in Fig. 11, clusters 1 and 3, was that DAP found in WTi/WT were not found in HOi/HO and vice versa. In other words, the response of signal transduction to infection was strongly affected by hemoglobin/phytoglobin overexpression.

### Polyamine and phytohormone signaling and metabolism

DAP associated with plant hormones grouped in two functional categories - in metabolism (where, in addition to hormones, there is also a polyamine subcategory) and in signal transduction (Supplementary Data Table S3). Because relatively few DAP were identified belonging to those categories, hormones and polyamines are shown together in Fig. 12, where part 1 comprises hormone-related DAP, and part 2 polyamine-related DAP. Among the hormone-related DAP are proteins taking part in ethylene biosynthesis – ACC oxidases (decreased abundance in HOi/WTi and increased in WTi/WT). DAP related to auxin (IAA) biosynthesis and perception decreased in abundance in HOi/HO or HOi/WTi or increased in WTi/WT. Two DAPs were related to abscisic acid (ABA) (decreased abundance in HOi/WTi), jasmonic acid (JA) (increased abundance in HOi/HO and WTi/WT), salicylic acid (SA) (increased abundance in HO/WT or WTi/WT) and brassinosteroids (BRs) (one DAP increased and one decreased in abundance in WTi/WT) (Fig. 12).

**Figure 13:**
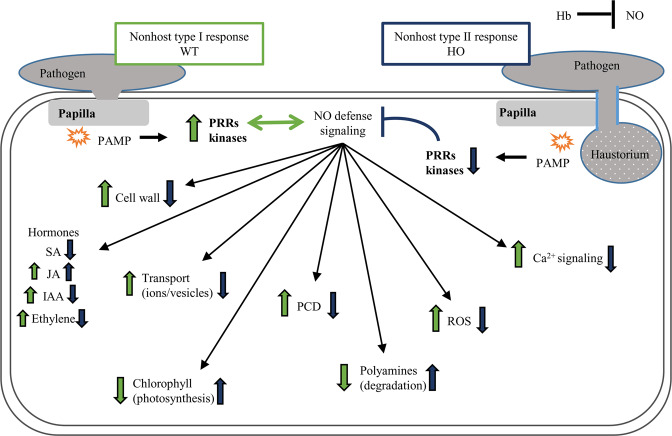
Schematic description of the changes observed in the proteome of barley plants during nonhost response. Arrows pointing upwards indicate a general trends of higher abundance of DAP associated with a given category, whereas arrows pointing downwards indicate a lower abundance. The changes in wild type (WT) are marked with green arrows, the plants with overexpression of phytoglobin (HO) are marked with black arrows.

NO signaling also interacts with plant hormone signaling^47^. RNA-binding protein BRN1-like (BAK01755.1) that acts as positive regulator of SA-mediated immunity and acts on SA signaling-related genes at a post-transcriptional level had a higher abundance only in WTi/WT (Fig. 13). The SA signal transduction pathway plays an important role in defense responses initiated by R‐genes^48^. However, its contribution to nonhost resistance is less clear. Arabidopsis mutants, which convert SA to catechol, and thus do not accumulate SA, were compromised in nonhost resistance to bacterial and fungal pathogens. However, this observation has not yet been confirmed for other plant species^49–51^.

The major polyamines (PA) in plants are the diamine, putrescine (Put), the triamine, spermidine (Spd) and the tetraamine, spermine (Spm). They function in key developmental and physiological events^52^ as well as taking part in plant response to stresses^53^. As for DAP associated with the metabolism of polyamines, the majority are involved in polyamine degradation, and their abundance increased in HOi/WTi. The exception was uridine 5’-monophosphate synthase (BAJ90460.1), which participates in the biosynthesis of the pyrimidine precursor. Its abundance increased in HOi/HO and in WTi/WT (Fig. 12).

NO in plants can be produced by reductive or oxidative pathways. Although oxidative pathways have not been fully elucidated, it has been established that the direct or indirect substrate in these reactions is L-arginine^54^. Arginine is the precursor of polyamines (PA) synthesis, connecting their metabolism with NO biosynthesis. Barley plants with overexpression of phytoglobins were also more resistant to drought, which was associated with increased polyamine production^55^. We observed an increase in the abundance of proteins responsible for the degradation of PA (flavin containing polyamine oxidase, and polyamine oxidase) (Fig. 12) in HOi/HO compared to WTi/WT, which suggests that polyamines are involved in the nonhost response of plants to pathogen attack and could play an important role. PAO-mediated Spm oxidation contributes to the onset of both host and nonhost HRs triggered in tobacco plants by different pathogens, highlighting the importance of Spm catabolism in the regulation of the HR-dependent defense response^56^. It would be interesting to examine PA in this context more closely in the future.

## Discussion

### The role of preinvasion resistance

Compared with 2-DE, high-throughput quantitative proteomics provides more powerful data for observation of protein expression profiles in different physiological processes in response to fungal attack. Until now, research has mainly focused on the study of proteome changes in compatible interactions^57–59^, but we need to know how nonhost resistance works to limit the destructive yield loss caused by powdery mildew. Two of most important mechanisms that cereals use to defend against nonhost *B. graminis* are the formation of papillae (cell wall appositions deposited on the inner surface of epidermal cell walls directly beneath aspersoria) (Fig. 8) and the hypersensitive reaction (HR) leading to PCD of attacked cells. The effective papilla present a physical and/or chemical penetration barrier^60^. Considering the main function of the phytoglobin in scavenging NO^61^, the observed differences between HO and WT plants may result from the difference in NO content. Overexpression of phytoglobins led to lower NO content both in Arabidopsis plants^62^ and in barley^55^. NO signaling also interacts with signaling by reactive oxygen species (ROS), calcium ions, kinases and hormones^47,63,64^. It was reported^8^ that the H_2_O_2_ content in barley WT plants increased after infection with *B. graminis* f. sp. *tritici* (H8), while that was not the case for the line overexpressing phytoglobin. Here we found a significant increase in several proteins associated with the organization of the cell wall in HO plants compared to WT, some of them also maintained an increased level after inoculation (Fig. 7, cluster 2). Upregulation of proteins connected to structural organization of cell wall can be associated with higher rate of papillae formation after inoculation in WT. In compatible interaction of barley with *B. graminis* f. sp. *hordei* the composition of papillae was found to not only consist of (1,3)‐β‐glucan (callose), but also heteroxylans, cellulose, phenolics (lignin and phenolic conjugates), arabinogalactan proteins, antimicrobial components, inorganic elements and ROS^65^. NO and ROS are not only necessary for papillae formation, but they also take part in signaling network leading to HR as a part of integrated defense system that involves phytohormones, activation of ion fluxes, changes in protein phosphorylation patterns, extracellular pH, membrane potential, oxidative cross-linking of plant cell wall proteins, and perturbations in the level of cytosolic Ca^2+^ ^44^. Thus, the differences in the penetration of barley cells by *B.graminis* f. sp. *tritici* noted by Sørensen *et al*.^8^ in both genotypes are consistent with changes in the abundance of proteins associated with preinvasion resistance related to subcategory of defense (Fig. 5), cell wall organization (Fig. 7), ion and vesicle transport (Fig. 9), and secondary metabolism (Fig. 10). This implies that NO plays an important role in these early stages of plant response to a pathogen attack and that in plants with overexpression of Hb, removal of NO does not lead to inhibition of next stages of defense that stop the pathogen.

### The role of signal transduction

Successful recognition of pathogens is one of the most important aspects of plant immunity as it leads to activation of all responses. Pattern recognition receptors (PRRs) recognize pathogen-associated molecular patterns (PAMPs) and initiate PAMP-triggered immunity (PTI). All known plant PRRs are plasma membrane-localized receptor-like kinases (RLKs) or receptor-like proteins (RLPs) with modular functional domains^66^. In our study after the inoculation more than 30 kinases increased in abundance in WT plants, and only three in HO plants. We also identified >10 kinases that increased in abundance in both genotypes in response to infection (Fig. 11, cluster 1 and 2). Activation of PRRs as well as the mitogen-associated and calcium-dependent protein kinases (MAPKs and CDPKs), leads to massive transcriptional reprogramming that is essential for PTI. The observed decreased kinase abundance in HOi may suggest that these signal pathways were not set in motion. Moreover, since proteins related to calcium signaling were only upregulated in WTi/WT (Fig. 11), we can conclude that the overexpression of phytoglobins somehow disturbs signal transduction. This may be caused by the ability of NO to activate MAPKs and the expression of defense genes. Salicylic acid-induced protein kinase (SIPK) may be activated by NO donors in *Nicotiana benthamiana*^67^ and recombinant NO synthases (from rat neuronal NOS) in tobacco^68^. How PAMP perception is linked to cytosolic Ca^2+^ elevation remains elusive. In *A. thaliana* secreted peptides that bind redundant LRR-RPKs activate its guanylyl cyclase activity to induce CNGC2-dependent Ca^2+^ rise. However, FLS2 (bacterial flagellin PRR) and EFR (bacterial elongation factor EF-Tu receptor) induce anion channels that requires Ca^2+^ channel activity independently of CNGC2, suggesting the involvement of additional Ca^2+^ channels in PAMP signaling^69,70^, which may be connected to NO signal transduction.

In addition to proteins associated with the turnover of the plant hormones IAA, ABA or CK, we also observed changes in the proteins of ethylene biosynthesis (Fig. 12). Ethylene biosynthesis involves two key steps: the conversion of *S*-adenosyl-L-Met to 1-aminocyclopropane-1-carboxylic acid (ACC) by ACC synthetase and then the oxidative cleavage of ACC to form ethylene. ACC oxidase catalyzes the conversion of ACC, ascorbate, and O_2_, to five products that are ethylene, cyanide, dehydroascorbate, CO_2_, and H_2_O, and it is the rate limiting step in ethylene biosynthesis^71^. Interestingly, barley showed a decreased abundance of ACC oxidase in HOi/WTi, but an increased abundance in WTi/WT (Fig. 12). This may mean that the production of ethylene was disturbed in the HO barley plants. The ethylene biosynthetic pathway could be regulated by mitogen activated kinases (MPK3 and MPK6) which in Arabidopsis phosphorylate ACC synthase, leading to stabilization and increased ethylene biosynthesis. In *mpk3,mpk6* double mutant plants, ethylene production in response to *Botrytis cinerea* infection was greatly reduced^72^. Lower induction of MAPKs in HOi plants could be a reason why the ACC synthase was not induced. Ethylene seems to inhibit symptom development in necrotrophic pathogen infection but enhances the cell death caused by other types of pathogens. Arabidopsis protoplasts isolated from the *etr1–1* mutant displayed reduced cell death from the fungal toxin fumonisin B1, and presence of the *ein2* mutation reduced cell death in the mutant characterized by accelerated PCD, supporting a role for ethylene in the regulation of programmed cell death^73^. Because of the conflicting information about HR in HO barley plants, it would be worth examining these issues further. But taking into account the reports of Sørensen *et al*.^8^, the change in abundance of vaculoar procesing 4 protein, MLO-like 15 and the disturbances in ethylene biosynthesis pathway in plants with overexpression of phytoglobins, they may show a time shift during induction of PCD in relation to the WT plants.

Little is known about the mechanisms of nonhost resistance even though it has been a topic of interest to plant pathologists for many years. This work identifies changes in the abundance of proteins that are components of different molecular machinery in relation to a normal nonhost response and a nonhost response weakened by phytoglobin overexpression. All changes observed in the plant proteome with overexpression of phytoglobins (HOi/HO) in comparison to the wild type after inoculation (WTi/WT) testify to a change in the type of response to a pathogen attack, however, without breaking immunity (Fig. 13). We wish to emphasize, that while our results give a detailed snapshot of the nonhost response after 72 hai, it is likely that sampling at earlier or later time-points will give complementary information about the plant-pathogen interaction. However, we have demonstrated that it is now possible to use quantitative proteomics to achieve a more global insight into the complex events of plant-pathogen interaction. Quantitative proteomic profiling can be used to understand the molecular background for observed physiological changes and be used as screening to identify new lines of research that will help us explain why some plants are resistant while others are not. One of the most important shifts in the type of nonhost resistance could be connected to papillae formation and disturbances in signal transduction, which are the result of NO and ROS disturbances. Further research using plants with overexpression of phytoglobins in nonhost response will be extremely useful for determining the more specific contribution of NO and phytoglobin in this type of immunity. This would improve our understanding of nonhost resistance, of the dynamics of plant disease resistance in general and, in the long term, the potential to genetically engineer plants for resistance against a broad range of pathogens.

## Materials and methods

### Plant material

Two barley (*Hordeum vulgare* L.) genotypes were used in the experiments, wild type plants of cultivar Golden Promise (designated as WT) and transgenic Golden promise lines (designated as HO) overexpressing cDNA of the barley phytoglobin/hemoglobin gene HvHb1 (accession number: U94968) controlled by the maize ubiquitin2 promoter. The transgenic line has previously been described^17^. Barley plants were grown for 16 days (second leaf fully emerged) in greenhouse cabins in 7 × 7 cm pots filled with a standard peat-based mixture of soil (Pindstrup Mosebrug A/S, Denmark). The greenhouse conditions were a temperature of 17 °C/day and 12 °C/night with 18 h of natural light supplemented with artificial light of 50–100 µmol m^−2^ s^−1^ when light outside was <10 000 lux.

### Pathogen material

The wheat-adapted isolate H8 of *Blumeria graminis* (DC.) Speer f. sp. *tritici* was used for inoculation. The isolate was kept and multiplied on seedlings of wheat (*Triticum aestivum* L.) cultivar Anja. Wheat seedlings were inoculated when they were 10 days old by dusting with spores from stock plants. The plants were then incubated in a dark cold room at 10 °C for 24 h and afterwards moved to spore-proof greenhouse cabins under the same conditions as described for barley plants. The infected wheat seedlings were covered with cellophane bags 4 days after inoculation and used for experimental inoculations when the infections were 14 days old. The infected plants were shaken 3 days before the inoculation of experimental barley plants to obtain fresh spores for the experiment.

### Infection procedure and sampling

The second leaves of 16-day-old barley seedlings were fixed on pedestals and inoculated with spores as described^8^. At least nine healthy leaves of WT and HO were inoculated, and the same number of leaves were mock treated (uninoculated control). Plants were inoculated with a spore density of app. 30 spores/mm^2^. Both mock and inoculated plants were incubated and then kept in the same spore-proof greenhouse cabin. A 2-cm piece was cut from the central part of all leaves 72 h after inoculation (hai). Leaves pieces from three different plants in separate pots were placed in vials and frozen immediately in liquid nitrogen as one biological replicate, resulting in a total of 3 replicates for each of the four treatments: mock-treated wild type plants (WT), mock-treated overexpression plants (HO), inoculated wild type plants (WTi) and inoculated overexpression plants (HOi).

### Proteomics

Optimized methods for protein extraction, digestion and TMT labelling have been developed for barley leaves by comparing a number of published methods^74^.

#### Protein extraction and digestion

Frozen leaves (0.45 g) were ground in liquid nitrogen, and proteins were extracted using extraction buffer: 2% (w/v) sodium deoxycholate (SDC), 10 mM dithiothreitol (DTT), polyvinyl polypyrrolidone (PVPP), 0.1 M triethylammonium bicarbonate (TEAB, pH 8.5), protease inhibitors (Complete™, EDTA free protease inhibitor cocktail, Roche) and phosphatase inhibitors (PhosSTOP™, Roche). The homogenate was incubated at 80 °C for 10 min and then sonicated in ice bath for 2 × 15 s with a 30 s break. Samples were vortexed vigorously at room temperature for 30 min, and then centrifuged for 15 min with 10 000 g and 15 min with 20 000 g. The amount of protein in the supernatant was quantified by amino acid analysis method^75^. Samples were prepared in three biological replicates and four conditions (two genotype types – WT and HO; inoculated and mock treatment). Protein digestion was carried out using the SDC-FASP protocol as described in^74^. A total of 100 µg protein was mixed with 200 µl 1% (w/v) SDC solution in 0.1 M TEAB, pH 8.5 and dialyzed in Microcon spin filter (10 000 g for 15 min at room temperature). Alkylation of free cysteines was performed on the filter membrane using 100 µl 0.05 M iodoacetamide in 1% SDC, 0.1 M TEAB solution, incubated for 30 min in dark at room temperature. To remove iodoacetamide, samples were centrifuged at 10 000 g for 15 min and washed twice with 1% SDC solution. Digestion of proteins was carried out in 50 µl of trypsin solution in 1% SDC, 0.1 M TEAB, pH 8.5 with an enzyme: protein ratio of 1:50 (w/w). Samples were incubated for 6 h at 37 °C. The peptides were collected by centrifugation and the filter was additionally washed with 1% SDC solution and combined with the peptide solution. SDC was removed from the samples by ethyl acetate extraction from the acidified solution using the phase transfer method^76^.

#### TMT labeling

Peptide samples were dried using a vacuum centrifuge and dissolved in 50 µl of 0.2 M TEAB. The pH of the solution was checked and adjusted to 8.0. The peptide concentration was determined by Qubit Protein Assay Kit (Thermo Fisher Scientific). Peptides (20 µg) were labeled with TMT 10 plex, using 8 tags (126, 127 C, 127 N, 128 N, 129 C, 129 N, 130 C and 130 N) according to the manufacturer’s protocol (Thermo Fisher Scientific). Three replicates of above four treatments were labeled with three series of the TMT tags: 126, 127 N, 128 N and 129 N for replicate one; 127 C, 129 C, 130 C and 30 N for replicates two; 126, 127 N, 128 N and 129 N for replicate three of each treatment. After labeling, replicates one and two and replicates two and three were mixed into two sets of TMT labels and desalted with Poros®20 R2 reversed phase microcolumns.

#### 2D-LC-MS/MS analysis

TMT labelled samples were analyzed by 2D-LC-MS, using high pH reverse phase (RP) fractionation as a first dimension followed by low pH RP chromatography, coupled to MS. For high pH RP fractionation samples were separated on an Ultimate3000 HPLC system (ThermoScientific) using ACQUITY CSH C18 1.7 µm column (300 µm × 100 mm) (Waters) with a linear gradient from 2 to 60% of buffer B in 1 h. Buffer A was H_2_O, 20 mM ammonium formate, pH 9; buffer B was 80% acetonitrile (ACN), 20% buffer A. 10 subfractions were collected (6 min per fraction) and combined into 5 fractions, thus fraction 1 contained subfractions 1 and 6, etc. The fractions were dried out in a speed-vac and resolubilized in 2% ACN, 98% H_2_O, 0.1% trifluoroacetic acid (TFA).

Full fraction volume after resolubilization was injected on the second dimension LC-MS on a Ultimate3000 RSLCnano HPLC system connected to a QExactive HF MS system (ThermoScientific). Samples were loaded on a cartridge precolumn PepMap 100 5*0.3 mm (ThermoScientific) in 2% ACN, 98% H_2_O, 0.1% TFA at 10 ml/min and then separated on a 1 m length 150 µm ID column, home-packed with InertSil ODS-3 2 µm sorbent (GLSciences)^77^. Separation was performed with a gradient of ACN, 0.1% FA (buffer B) in H_2_O, 0.1% FA (buffer A) from 4 to 32% buffer B in 2 h at 0.7 µl/min at 55 °C.

MS analysis was carried out in a DDA mode with 1 MS1 scan, followed by TOP20 MS2 scans. MS1 parameters were 120000 resolution, 3e6 AGC target, maximum IT 100 ms, scan range 300 to 2000 m/z. MS2 parameters were 60000 resolution, 2e5 AGC target, maximum IT 108 ms, isolation window 1.2 m/z, isolation offset 0 m/z, fixed first mass 110 m/z, (N)CE 30, minimum AGC 8e3, exclude unassigned and 1, 6–8 charges, preferred peptide match, exclude isotopes ON, dynamic exclusion 40 s.

### Protein identification and quantification

Raw MS/MS data were processed using Proteome Discoverer 2.1 (Thermo Scientific) and searched using the Mascot search engine. The two fractionated TMT sets were searched through the processing workflow in batch mode, followed by a multiconsensus workflow combining the files. The Mascot parameters for protein identification were defined as follows: database – NCBI^78^, *Hordeum vulgare* protein database (updated on 18 October, 2018); precursor mass tolerance – 20 ppm; fragment mass tolerance – 0.05 Da; digestion – trypsin with two missed cleavages allowed; fixed modification: Carbamidomethyl (C), TMT6plex (K) and TMT6plex (N-term); variable modification: methionine oxidation (M). The Percolator was used for peptide validation. Protein quantification was performed using the Reporter Ion Quantifier node (processing workflow) to estimate the peak intensity of the reporter ions, followed by the use of the Peptide and Protein Quantifier (consensus workflow) node embedded in Proteome Discoverer 2.1, where protein abundance is calculated as the average of the three most abundant distinct peptides identified for the protein. A cut-off value of at least one unique peptide per protein was applied. The list of all identified and quantified proteins can be found in Supplementary Table S1. The mass spectrometry proteomics data have been deposited in the ProteomeXchange Consortium via the PRIDE^79^ partner repository with the dataset identifier PXD015089 (Reviewer account details: Username: reviewer62787@ebi.ac.uk; Password: gDwPvDIk).

We checked whether it was possible to detect fungal proteins in the samples. After the identification of plant proteins, all peptides with lower than high confidence were checked against a fungal database – UniProt^80^, *Blumeria graminis* f. sp. *tritici* (updated on 4 October, 2018) with search parameters similar to the plant proteome analysis. Only 7 fungal proteins were identified, but all of them were present in both mock and infected samples. For this reason they were treated as a plant proteins with a conserved sequence and similar to fungal proteins and not analyzed further. The list of proteins is found in Supplementary Table S5.

### Statistical analysis

Identified protein from Proteome Discover 2.1 were analyzed using LimmaRP^20^. For differentially accumulated proteins (DAP), a q-value below 0.05 was considered statistically significant for two-group comparisons. sPLS – DA was done using MetaboAnalyst 4.0. To visualize the differences between all factors, Venn diagram and heatmaps were constructed using R version 3.5.1 - “Feather Spray” and packages: VennDiagram^81^ version 1.6.0, ComplexHeatmap^82^ version 1.18.1. K-means clustering was done with pre-defined Euclidean method available in R package, heatmaps where then split in two, three or four groups for easiest comparison.

## Supplementary information

Supplementary Table S1

Supplementary Table S2

Supplementary Table S3.

Supplementary Table S4.

Supplementary Table S5

Supplementary Figure S1
