# Supplementary figures and images for: The effect of phytoglobin overexpression on the plant proteome during nonhost response of barley (Hordeum vulgare) to wheat powdery mildew (Blumeria graminis f. sp. tritici)
Source: Sci Rep. 2020 Jun 8;10:9192. doi: 10.1038/s41598-020-65907-z (PMC7280273; doi:10.1038/s41598-020-65907-z)

**A**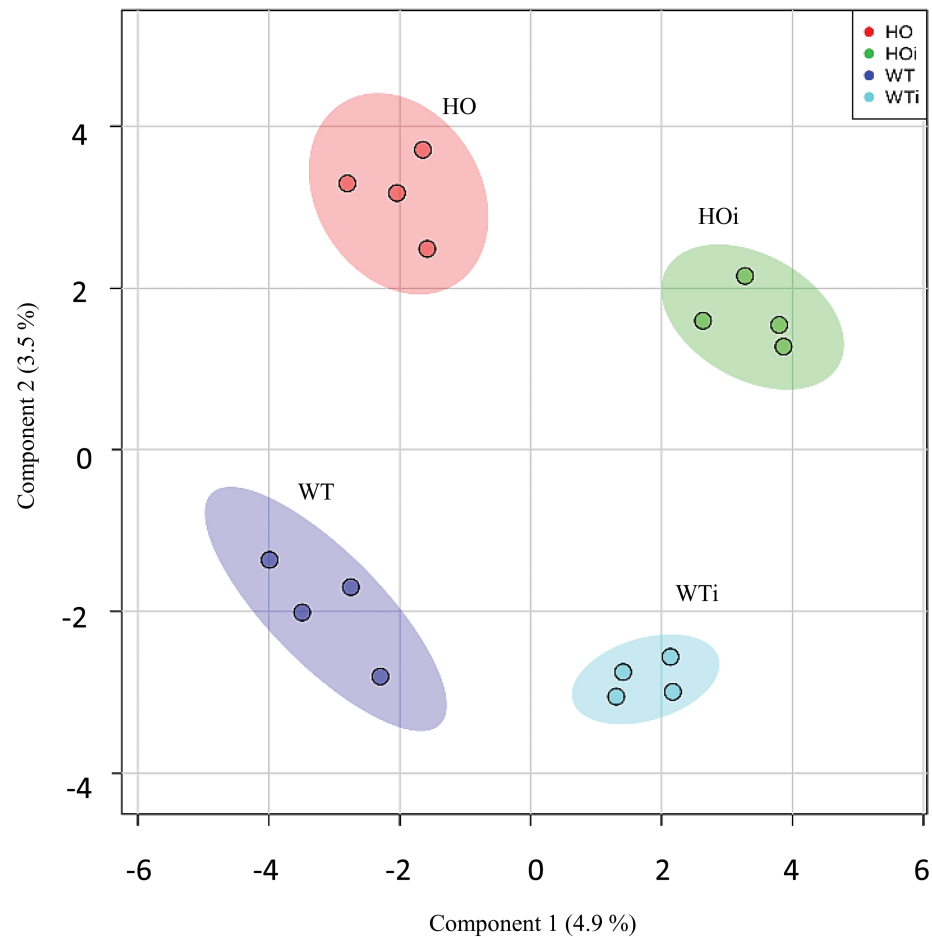**B**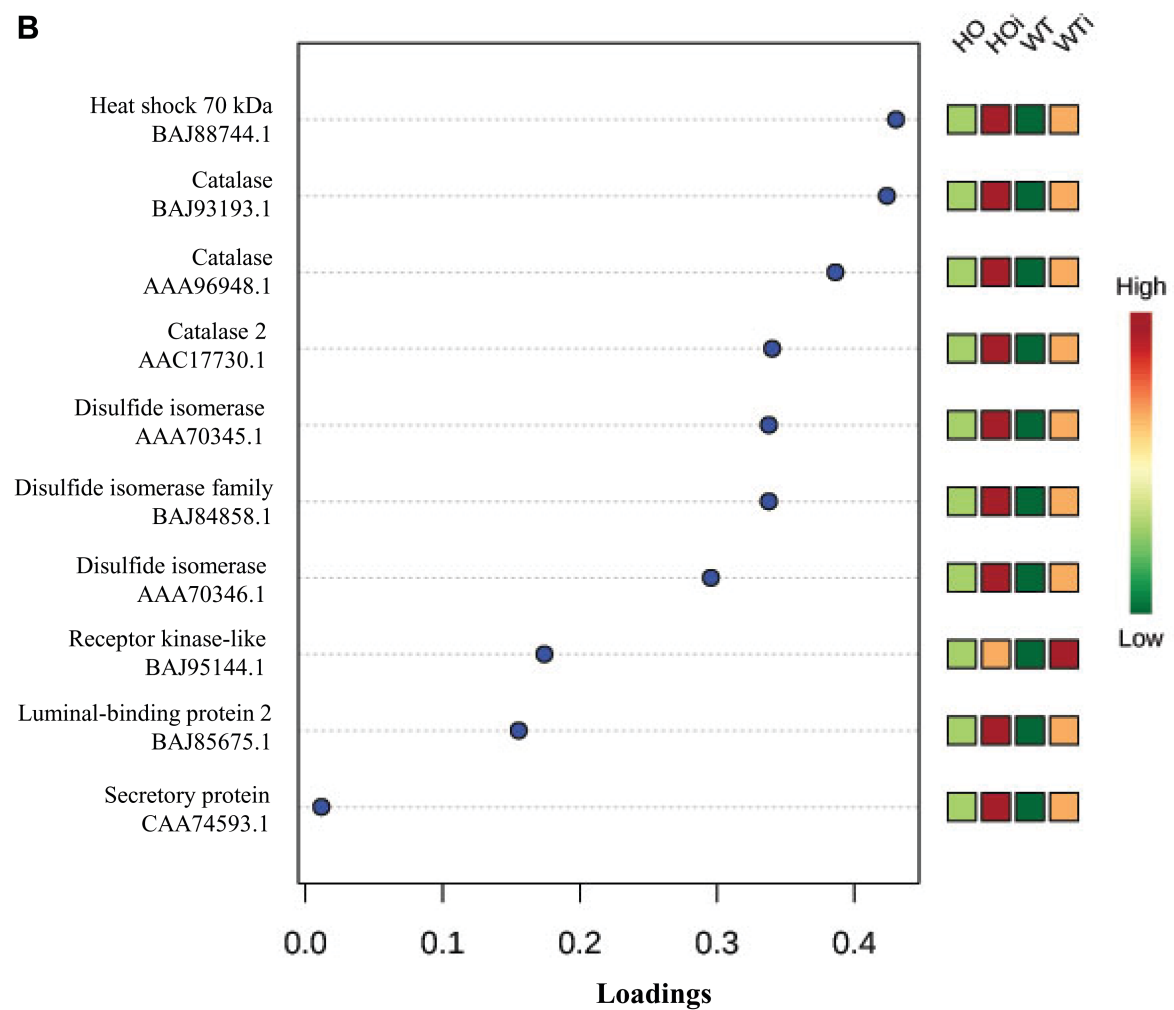

Supplement: Supplementary file 6 — Supplementary Figure S1 [file 41598_2020_65907_MOESM6_ESM.pdf]
